# Surface display of recombinant proteins on *Escherichia coli* by BclA exosporium of *Bacillus anthracis*

**DOI:** 10.1186/1475-2859-12-81

**Published:** 2013-09-22

**Authors:** Tae Jung Park, Nam Su Heo, Sung Sun Yim, Jong Hyun Park, Ki Jun Jeong, Sang Yup Lee

**Affiliations:** 1 Department of Chemistry, Chung-Ang University, 84 Heukseok-ro, Dongjak-gu, Seoul 156-756, Republic of Korea; 2 BioProcess Engineering Research Center, 291 Daehak-ro, Yuseong-gu, Daejeon 305-701, Republic of Korea; 3 Department of Chemical & Biomolecular Engineering (BK21), 291 Daehak-ro, Yuseong-gu, Daejeon 305-701, Republic of Korea; 4 Department of Bio & Brain Engineering, Department of Biological Sciences, KAIST Institute for the BioCentury, Center for Systems & Synthetic Biotechnology, and Bioinformatics Research Center, KAIST, 291 Daehak-ro, Yuseong-gu, Daejeon 305-701, Republic of Korea

## Abstract

**Background:**

The anchoring motif is one of the most important aspects of cell surface display as well as efficient and stable display of target proteins. Thus, there is currently a need for the identification and isolation of novel anchoring motifs.

**Results:**

A system for the display of recombinant proteins on the surface of *Escherichia coli* was developed using the *Bacillus anthracis* exosporal protein (BclA) as a new anchoring motif. For the surface display of recombinant proteins, the BAN display platform was constructed in which a target protein is linked to the C-terminus of N-terminal domain (21 amino acids) of BclA. The potential application of BAN platform for cell surface display was demonstrated with two model proteins of different size, the *Bacillus* sp. endoxylanase (XynA) and monooxygenase (P450 BM3m2). Through experimental analysis including outer membrane fractionation, confocal microscopy and activity assay, it was clearly confirmed that both model proteins were successfully displayed with high activities on the *E. coli* cell surface.

**Conclusions:**

These results of this study suggest that the strategy employing the *B. anthracis* BclA as an anchoring motif is suitable for the display of heterologous proteins on the surface of *E. coli* and consequently for various biocatalytic applications as well as protein engineering.

## Background

Cell surface display allows expression of proteins or peptides on the surface of cells in a stable manner using the surface proteins of bacteria, yeast, or even mammalian cells as anchoring motifs [1-4]. This powerful tool has been used in a wide range of biotechnological and industrial applications, such as live vaccine development [5], peptide libraries screening [6,7], whole-cell catalysis [8], biosensor development [9,10] and environmental bio adsorption [11,12]. For the efficient display of recombinant proteins on a surface of host cells, various anchoring motifs have been developed, including OprF, OmpC, OmpX, and many others [12-14]. Although many successful results have been achieved, the use of current anchoring motifs did not always allow efficient display of all target proteins [3]. In cell surface display systems, successful protein display is highly dependent on the choice of the anchoring motif. Therefore, in this study, we decided to explore and develop an alternative cell surface display system for the expression and display of recombinant proteins.

**Figure 1:**
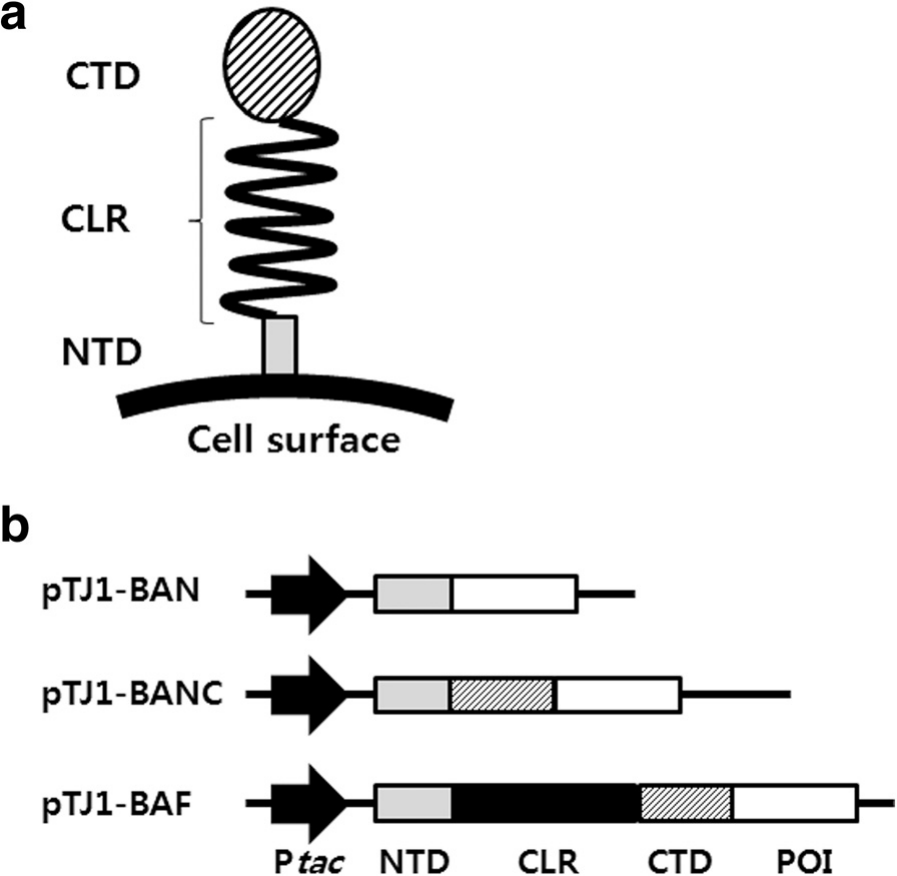
**Cell surface display system using the BclA anchoring motif. (a)** Schematic illustration of BclA on the bacterial surface. NTD, N-terminal domain of BclA; CLR, GXX triplet repeated collagen-like region; CTD, C-terminal domain of BclA. **(b)** The structure of the constructed surface anchoring vectors (pTJ1-BAN, pTJ1-BANC and pTJ1-BAF) with the domains of the BclA protein (NTD, gray box; CLR, black box, CTD, hatched box) and target protein (white box). P*tac*; *tac* promoter, POI; protein of interest.

The surface protein to be used as an anchoring motif should possess in general an efficient signal sequence to facilitate the translocation of a foreign protein through the inner membrane of the cell, a targeting signal for anchoring a foreign protein to the surface of the cell in a stable manner, and accommodating foreign proteins or peptides of various sizes. Furthermore, the fusion protein should be expressed in large amounts [2-4]. Here, we developed a cell surface display system using the *B. anthracis* BclA as a potential anchoring motif. The BclA is an exosporium protein, a hair-like protein surrounding the *B. anthracis* spore. The *B. anthracis* BclA proteins were found to possess conserved amino acid sequences (red-colored in Additional file 1: Figure S1a) in the N-terminal domain (NTD), C-terminal domain (CTD), and central (GPT)_X_GDTGTT triplet repeating region (blue-colored in Additional file 1: Figure S1a) making a collagen-like structure (Figure 1a) [15,16]. In this work, we developed a protein display system (BAN platform) using the NTD (21 amino acids) of BclA as an anchoring motif and its display efficiencies were examined with two model recombinant proteins, a relatively small *Bacillus* sp. endoxylanase (XynA, 21.2 kDa) and a much bigger *Bacillus megaterium* monooxygenase (P450 BM3m2, 120 kDa).

## Results

### Development of a surface display system with BclA

The native BclA contain 19-residue amino terminal peptide, but this peptide is proteolytically removed during sporulation and the remaining mature BclA is attached to the surface of the developing forespore [15,16]. The mature BclA protein consists of three parts: NTD, CTD and the central domain, which contains 1 ~ 8 repeating regions of ‘(GPT)_X_GDTGTT triplet sequence’ (Figure 1a and Additional file 1: Figure S1) [15,16]. The central domain looks like a mammalian collagen protein (it is also called ‘collagen-like region’, CLR) and, according to the repeating number of CLR, the BclA can be of different sizes (253 ~ 445 amino acids). Each domain has its own unique independent function. In addition, the truncated form of each domain shows different levels of expression and localization on the membrane [8,16,17]. Thus, in order to develop the most efficient display system based on the *B. anthracis* BclA, we examined three different display systems using the three different motifs (BAN, BANC, and BAF) of BclA as anchoring motifs as shown in Figure 1b. The BAN system contains only the NTD (21 amino acids) without the 19-residue amino-terminal peptide of BclA, and the BANC system contains both NTD and CTD without the central CLR (total 178 amino acids). Finally, the BAF system contains a mature form of full-length BclA from *B. anthracis* RA3 strain (NCBI accession no. CAD56878.1, 233 amino acids). In each system, the target protein (*Bacillus* sp. TG43 lipase) was fused to the C-terminus of each anchoring motif, and gene expression was controlled under the IPTG-inducible *tac* promoter (P_*tac*_). Comparison of gene expression in three systems suggested that the BAN expression platform (pTJ1-BAN) allowed significantly higher gene expression, while other expression systems (BANC and BAF) showed rather poor expression levels (Additional file 2: Figure S2). Even though much higher production level could be achieved with the BAN-fused system, the localization of BAN-fused lipase on cell surface gave multiple varying results during the repeated experiments. Thus, the display of other recombinant proteins instead of lipase was studied using the BAN platform as a display system in further experiments.

### Display of endoxylanase on the *E. coli* cell surface

**Figure 2:**
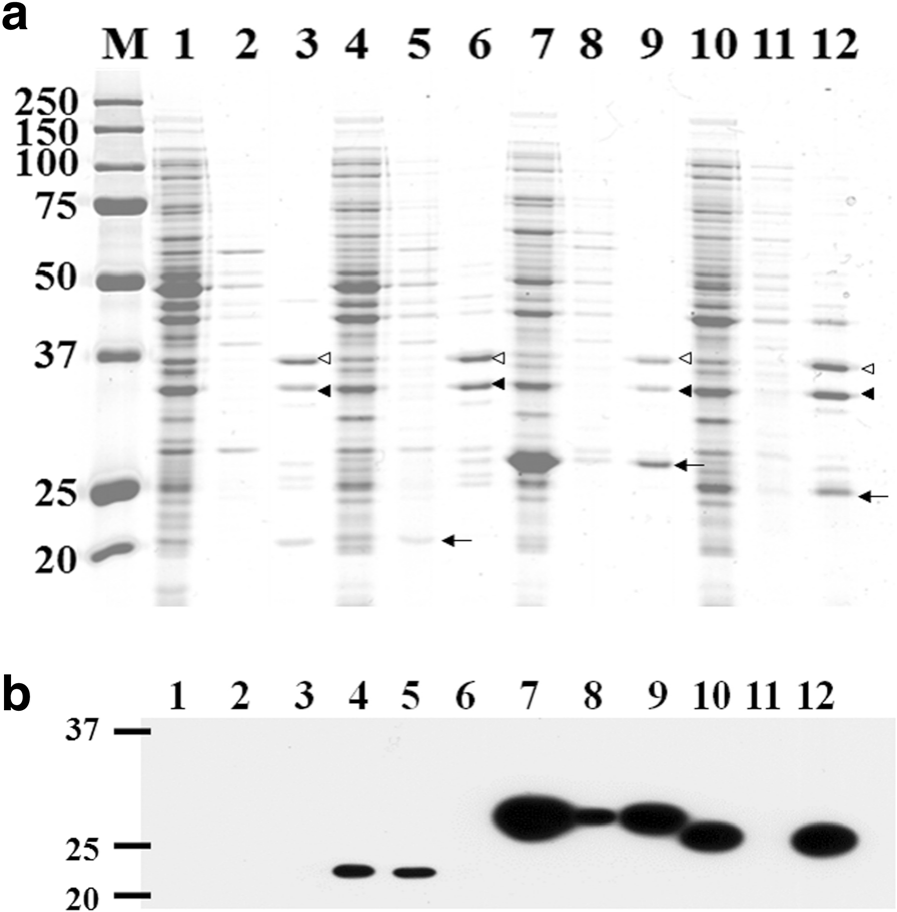
**Display of endoxylanase on cell surface. (a)** SDS-PAGE analysis and **(b)** western blotting analysis of *E. coli* displaying BAN-fused XynA. Lane M, molecular weight size markers; Lanes 1, 2 and 3, *E. coli* host only without plasmid; Lanes 4, 5, and 6, *E. coli* harboring pTJ1-pelB-XynA; Lanes 7, 8, and 9, *E. coli* harboring pTJ1-BAN-XynA; Lanes 10, 11, and 12, *E. coli* harboring pTJ1-pelB-BAN-XynA. Lanes 1, 4, 7, and 10, total proteins; Lanes 2, 5, 8, and 11, periplasmic protein fraction; Lanes 3, 6, 9, and 12, outer membrane fraction. Arrows in lanes 5, 9 and 12 indicate XynA (~21 kDa), BAN-XynA with His6 tag (~27 kDa), BAN-XynA (~25 kDa) and, respectively. Closed and open arrowheads indicate the bands of OmpA and OmpC proteins in outer membrane proteins fraction, respectively.

**Figure 3:**
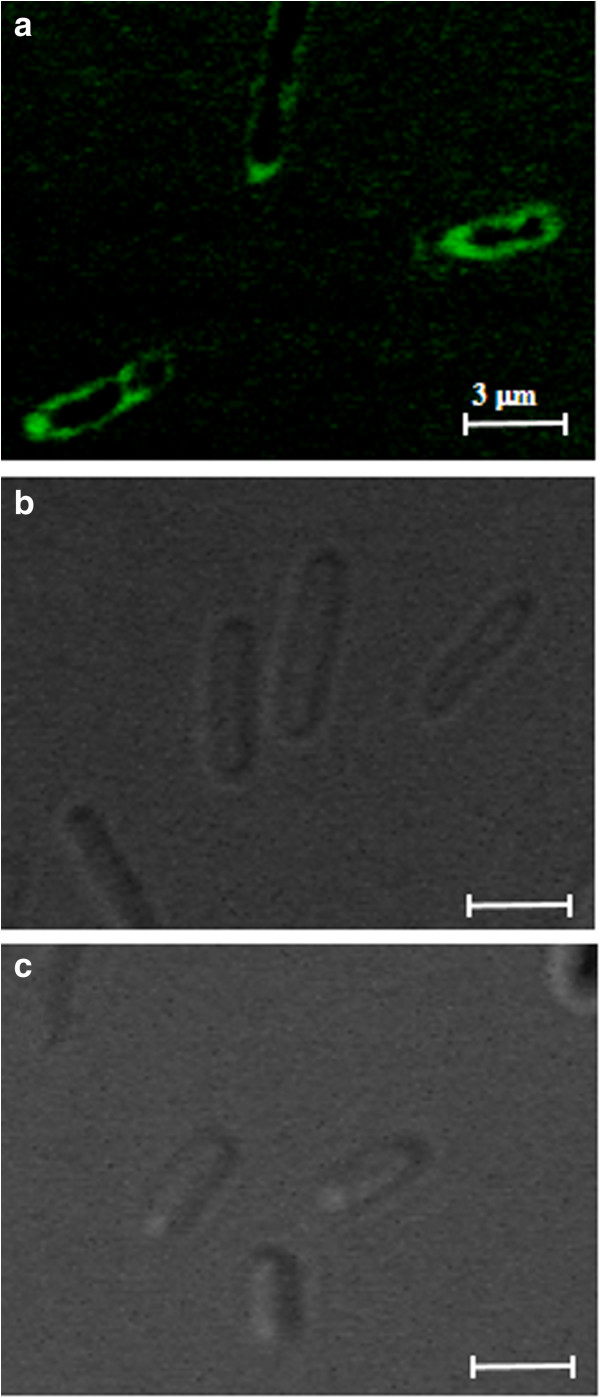
**Confocal immunofluorescence microscope image. (a)***E. coli* harboring pTJ1-BAN-XynA, **(b)***E. coli* harboring pTJ1-pelB-XynA, and **(c)***E. coli* host only without plasmid. Each scale bar represents 3 μm.

To demonstrate the potential of BclA as an anchoring motif for cell surface display, the *Bacillus* sp. endoxylanase (~21.2 kDa), which can hydrolyze xylan to xylooligosaccharides (xylose, xylobiose, xylotriose, etc.) [18], was examined. For the production of BAN-fused endoxylanase, two plasmids, pTJ1-BAN-XynA and pTJ1-pelB-BAN-XynA, were constructed in which the BAN motif was fused to XynA without or with the PelB signal peptide, respectively. As a negative control, pTJ1-pelB-XynA, which does not contain the BAN anchoring motif but containing the PelB signal peptide for the periplasmic production of XynA, was also constructed. Recombinant *E. coli* strains harboring pTJ1-pelB-XynA, pTJ1-BAN-XynA, and pTJ1-pelB-BAN-XynA were cultivated, and the localization of endoxylanase on the cell surface was analyzed by SDS-PAGE and western blotting. The BAN-linked endoxylanase was clearly detected in the total lysate and outer membrane fractions of both *E. coli* cells harboring pTJ1-BAN-XynA and pTJ1-pelB-BAN-XynA (Figure 2a). However, when the PelB signal peptide without the BAN motif (pTJ1-pelB-XynA) was used, endoxylanase was not detected in the membrane fraction, but was detected in the periplasmic fraction only (Figure 2a). The production and localization of BAN-fused endoxylanase on membrane fractions were also clearly confirmed by western blotting (Figure 2b). In addition, localization of endoxylanase could be confirmed by confocal microscopy. After cultivation, cells were labeled with the FITC-conjugated anti-FLAG antibody probe, which can recognize the FLAG tag linked to C-terminus of endoxylanase. *E. coli* cells harboring pTJ1-BAN-XynA and pTJ1-pelB-BAN-XynA showed the strong fluorescence, while *E. coli* harboring pTJ1-pelB-XynA did not show any fluorescence signal (Figure 3). This means that the BAN anchoring motif mediated the secretion and localization of XynA on the surface of *E. coli* independent of the signal peptide.

**Figure 4:**
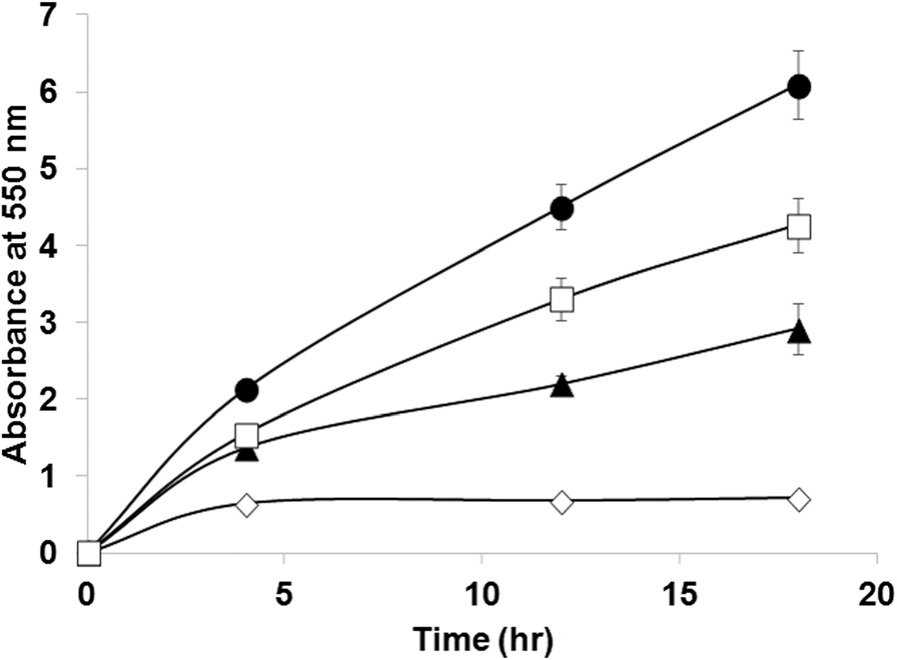
**Endoxylanase activity assay.** After flask cultivation, whole cells were used for activity assay of endoxylanase (XynA) on cell surface. Symbols: ◊, *E. coli* host only without plasmid; ▲, *E. coli* harboring pTJ1-pelB-XynA; □, *E. coli* harboring pTJ1-pelB-BAN-XynA; ●, *E. coli* harboring pTJ1-BAN-XynA.

The specific endoxylanase activities of whole cells were determined using beechwood xylan as a substrate. Immediately after mixing with xylan, *E. coli* harboring pTJ1-BAN-XynA also showed a significant increase in the concentration of reducing sugars compared with other controls (*E. coli* cells harboring pTJ1-pelB-XynA or pTJ1-pelB-BAN-XynA) (Figure 4). This result suggests that the endoxylanase was successfully anchored to outer membrane by fusion with BAN anchoring motif, and the displayed endoxylanase showed high activity. The use of PelB signal peptide with the BAN anchoring motif (pTJ1-pelB-BAN-XynA) also allowed the secretion and localization of the BAN-fused endoxylanase. However, its display efficiency and activity were relatively lower than those obtained with the BAN motif only. Thus, the use of signal peptide is not necessary in the BAN display platform.

The localization of excess proteins on outer membrane might cause problems in cell wall integrity, and consequently result in cell lysis and possible release of anchored proteins into the culture medium. Thus, the contamination of endoxylanase in the culture supernatant was analyzed during the display of endoxylanase on the surface. After cultivation of *E. coli* harboring pTJ1-BAN-XynA, the presence of endoxylanase on the surface and culture medium were analyzed by SDS-PAGE followed by western blotting analysis. The BAN-fused endoxylanase was clearly detected in the total cell lysate and membrane fractions, but not in the supernatant fraction (Additional file 3: Figure S3). This result indicates that BAN-fused endoxylanases were displayed on the surface and were not released into the culture medium. The specific endoxylanase activities in whole cells and culture supernatant were determined as described above. A low level of activity from the culture supernatant of *E. coli* (pTJ1-BAN-XynA) was obtained, but it was much lower than that of the whole-cell suspension (Additional file 4: Figure S4). This result also indicates that the BAN-fused endoxylanase was successfully anchored on the cell surface and was stably maintained without cell lysis.

### Display of monooxygenase on the *E. coli* cell surface

**Figure 5:**
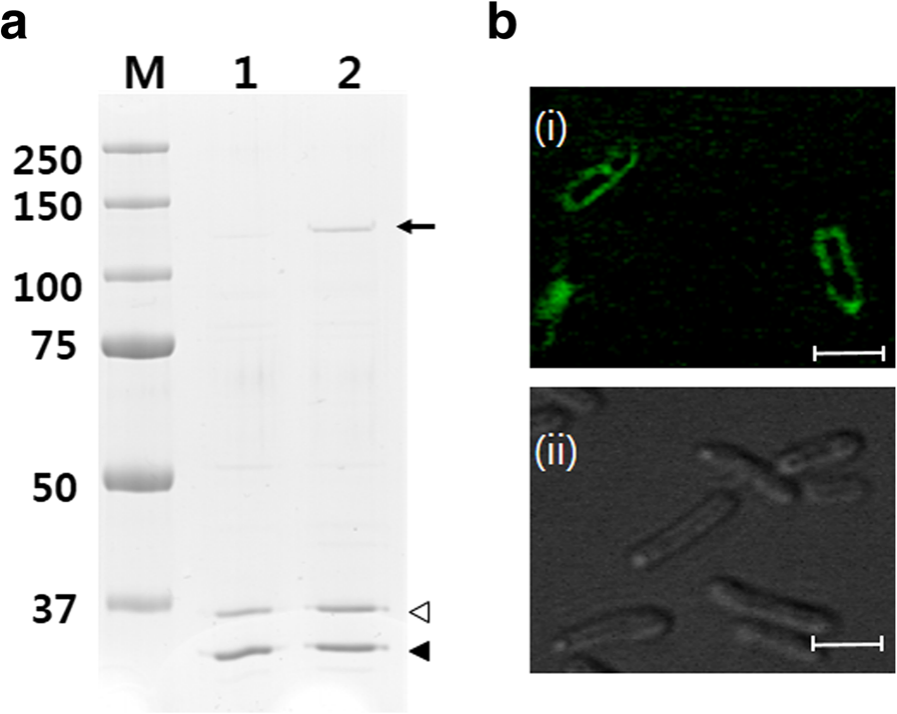
**Display of BAN-fused monooxygenase (P450 BM3m2) on the cell surface. (a)** SDS-PAGE analysis of *E. coli* displaying BAN-fused P450. Lane M, molecular weight size markers; Lanes 1 and 2, outer membrane proteins fraction of *E. coli* harboring pTJ1- BM3 or pTJ1-BAN-BM3, respectively. Closed and open arrowheads indicate the bands of OmpA and OmpC proteins in outer membrane proteins fraction, respectively. Arrow indicates BAN-BM3m2 (~120 kDa). **(b)** Confocal immunofluorescence microscope images. (i) *E. coli* harboring pTJ1-BAN-BM3 and (ii) *E. coli* harboring pTJ1-BM3. Cells were stained with FITC conjugated anti-FLAG-antibody. Each scale bar represents 3 μm.

**Figure 6:**
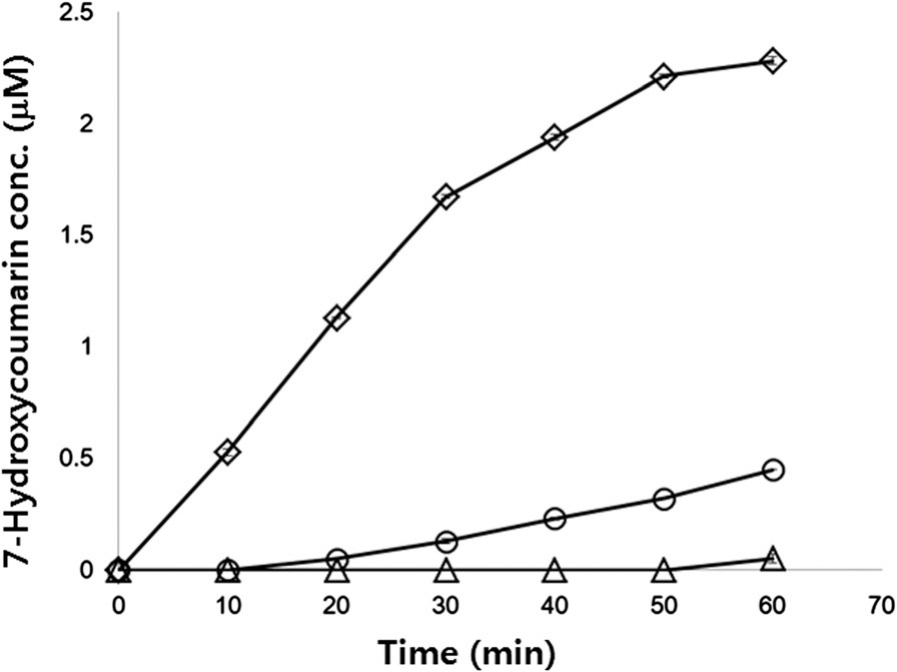
**Activity assays of monooxygenase (P450 BM3m2) displayed on the *E. coli *cell surface.** After the reaction with 7-epoxicoumarin, the concentration of product (7-hydroxycoumarin) were analyzed. Symbols: ◊, *E. coli* harboring pTJ1-BAN-BM3; ○, *E. coli* harboring pTJ1-BM3; Δ, *E. coli* harboring pTJ1-BAN.

To demonstrate the general use of the BAN anchoring motif for cell surface display, cytochrome P450 monooxygenase variant (P450 BM3m2) from *B. megaterium* was also examined; this protein is much bigger (ca. 120 kDa) than endoxylanase. After flask cultivation of *E. coli* XL1-Blue harboring pTJ1-BAN-BM3, localization of the BAN-fused P450 BM3m2 on the cell surface was confirmed by SDS-PAGE. The band of P450 BM3m2 (~120 kDa) was clearly detected on the Coomassie-brilliant blue stained SDS-PAGE gel (Figure 5a). However, with *E. coli* harboring pTJ1-BM3 in which the BAN motif was not used, P450 BM3m2 was produced in the cytoplasm only and was not detected in the outer membrane fraction. The localization of P450 BM3m2 on the cell surface was also confirmed by confocal microscopy. After cultivation and labeling with the FITC-conjugated anti-FLAG antibody probe which can recognize the FLAG tag linked to C-terminus of P450 BM3m2. *E. coli* harboring pTJ1-BAN-BM3 clearly exhibited strong fluorescent signals, but *E. coli* harboring pTJ1-BM3 did not show any fluorescent signals (Figure 5b). The activity of monooxygenase (P450 BM3m2) on the surface of *E. coli* cells was verified using a specific activity assay. In this activity assay, the catalytic conversion of 7-ethoxycoumarin to 7-hydroxycoumarin requires the regeneration of oxidative monooxygenase, and NADPH was externally supplied for this regeneration purpose. However, it is known that NADPH cannot penetrate the cell membrane [19], so only the monooxygenase displayed on the cell surface can utilize the supplied NADPH and continue the oxidative reaction. However, cytoplasmic monooxygenase cannot be regenerated due to the absence of supplied NADPH, and it cannot continue the reaction. The high activity of monooxygenase indicates the localization of monooxygenase on the cell surface not in the cytoplasm. Compared with the negative controls (pTJ1-BM3 and pTJ1-BAN), *E. coli* harboring pTJ1-BAN-BM3 showed much higher activity against the 7-ethoxycoumarin substrate (Figure 6). Therefore, the higher activity of cells harboring pTJ1-BAN-BM3 than that of pTJ1-BM3 (Figure 6) indicates that P450-BM3 was located on the surface not in the cytoplasm or periplasm.

## Discussion

Microbial cell-surface display systems can be used in a wide range of applications as described earlier. The display of active enzymes has been intensively pursued due to its potential use as a whole-cell biocatalyst in the fields of pharmaceuticals, fine chemicals and agrochemicals production. To date, several cell-surface display systems have been developed for the expression of polypeptides or proteins on the surface of *E. coli* and, as an anchoring motif, outer membrane proteins such as OmpC, OmpX, maltoprotein LamB, the outer membrane protein S, lipoprotein TraT and many others, have been employed, which were fused to the protein of interest [3]. In most of the surface display systems that use outer membrane proteins as anchoring motifs, target proteins are fused to the anchoring motif via a sandwich fusion format. This results in minimal destabilization of the anchoring motif on the outer membrane, and improved efficiency of surface display. In an alternative, which includes the OmpX case and has been called the circularly permutation strategy, both the N and C termini are presented on the external cell-surface [14]. However, in most cases, the passengers displayed have been limited primarily to short peptides or polypeptides. In the present study, the *B. anthracis* exosporal protein BclA was examined as a new anchoring motif. The C-terminal fusion strategy was employed to display foreign proteins. In this display format, both proteins (endoxylanase and monooxygenase) were successfully displayed with high activities. These results suggest that the C-terminal deletion-fusion strategy employing the *B. anthracis* BclA is suitable for the display of heterologous polypeptides and proteins, which should prove useful in a wide range of applications. Particularly, the successful display of monooxygenase (P450 BM3m2), which has much bigger size than the average size of passenger proteins [3], was very promising. In general, most cell display systems have size limitations in regards to the protein to be displayed, and most systems are only suitable for peptides or relative small polypeptides (below 50 kDa). So far, there is only one successful case for the display of P450 on a bacterial cell surface, where an ice-nucleation protein (INP) was used as an anchoring motif [19]. When other well-known anchoring motifs including OmpC and OmpX were tested, such a big-size enzyme (P450 BM3m2) could not be displayed (data not shown). The successful display of P450 using the BAN anchoring motif clearly indicates the potential of the BAN anchoring motif to accommodate many different passenger proteins.

Although two model proteins of different sizes could be successfully displayed on the cell surface using the BclA anchoring motif, the mechanisms for the secretion and anchoring of BclA and BclA-fused proteins are unknown at this time. In general, the protein secretion into periplasm and cell surface including cell surface display requires a signal peptide. The native BclA protein synthesized in the mother cell is attached to the surface of the forespore; the 19-residue amino-terminal peptide is proteolytically removed and the next 20–40 amino acids (NTD) are used for anchoring. Also, it is known that the anchoring of the mature BclA protein on the surface of *B. anthracis* requires a few specific exosporium receptor proteins (i.e., BxpB or its homologue ExsFB) [20-22]. In the display system reported here, only the NTD (21 amino acids) after the first 19 amino acids of BclA was employed. To our knowledge, the receptor proteins reported for *B. anthracis* are not produced in *E. coli*. As demonstrated with two model proteins (endoxylanase and P450 BM3m2), their fusions with the BAN motif resulted in their successful secretion and localization onto the cell surface, which was clearly confirmed by several experiments including membrane protein fractionation, confocal microscopy, and activity assay. The use of the well-known PelB signal peptide for the secretion of BAN-fused proteins also allowed the localization of proteins on the cell surface of *E. coli*, but the efficiencies were relatively lower than that obtained with the signal peptide-free system (Figures 2, 3 and 4).

Thus, it will be an interesting future study to decipher the detailed mechanisms for such signal peptide-free secretion using the BAN system. However, this is not the only case reported. The ice nucleation protein (INP) of *Pseudomonas syringae* has been used as a great anchoring motif for the cell surface display of various proteins in *E. coli* and other gram negative bacteria. The INP and its fusion proteins do not require any signal peptide for their secretion and anchoring on the cell surface [8,17,23]. Interestingly, the BclA protein has structural similarity to INP which also comprises three domains (NTD, CTD and CLR). Thus, although there is no proof that INP and BclA could mediate membrane transport and cell attachment in a similar way, the mechanism of BAN-based signal peptide-free secretion might be similar to that of INP. Further studies are needed to understand the mechanism.

## Conclusions

We developed a new cell surface display platform based on the BclA of *B. anthracis* as an anchoring motif. The C-terminal fusion of two model proteins to BclA (especially BAN) allowed their successful secretion and surface display with high efficiency and stability. Although only two proteins (21 and 120 kDa) were examined, the results seem to indicate the versatility of this display system capable of secreting and displaying proteins of different sizes; this is an important advantage of the BAN anchoring motif. Furthermore, both enzymes displayed using the BAN system, endoxylanase (XynA) and monooxygenase (P450 BM3m2), showed high activities and stabilities (as examined for xylanase). By displaying different enzymes of interest using our system, it will be possible to develop cost-effective bio catalytic systems in the fields of pharmaceuticals, fine chemicals, agrochemicals and other demanding industries.

## Materials and methods

### Bacterial strains and growth conditions

The bacterial strains and plasmids used in this study are summarized in Table 1. All *E. coli* cells were cultivated in Luria-Bertani (LB) medium (10 g/L of tryptone, 5 g/L of yeast extract, and 5 g/L of NaCl) supplemented with 50 μg/mL of ampicillin (Ap) at 37°C and 200 rpm. For the display of endoxylanase, cells were induced at an OD_600_ of 0.6 with 1 mM isopropyl-β-d-thiogalactopyranoside (IPTG). After induction, all cells were further cultured for 4 h and then harvested by centrifugation (10,000 × *g* for 10 min at 4°C) for further analysis. For the display of P450 BM3m2, *E. coli* cells were induced at the same cell density with 0.4 mM IPTG. In addition, 1 mM of thiamin and 0.5 mM of δ–aminolevulinic acid (Sigma-Aldrich, St. Louis, MO) as a heme precursor were added into each culture at the same time with IPTG induction. After further cultivation at 30°C for 6 h and 150 rpm, cells were harvested by centrifugation at 6,000 rpm for 10 min for further analysis.

**Table 1:** Bacterial strains and plasmids used in this study

**Strain or plasmid**	**Relevant characteristics**	**Reference or source**
**Strains**		
*E. coli* JM109	*F’traD36 proA + B + lacIq Δ(lacZ)M15/ Δ(lac-proAB) glnV44 e14- gyrA96 recA1 relA1 endA1 thi hsdR17*	New England Biolabs^a^
*E. coli* XL1-Blue	*recA1 endA1 gyrA96 thi-1 hsdR17 supE44 relA1 lac* [F’ *proAB lacI*q*Z*Δ*M15* Tn*10* (Tetr)].	Stratagene^b^
**Plasmids**		
pTrc99A	4.17 kb, *bla*, *trc* promoter	Pharmacia^c^
pTac99A	5.68 kb, pTrc99A derivative; *tac* promoter	[24]
pTJ1-BAN	5.76 kb, pTac99A derivative; N-terminal of *bclA*	This study
pTJ1-BANC	6.23 kb, pTac99A derivative; N- & C-terminal of *bclA*	This study
pTJ1-BAF	6.45 kb, pTac99A derivative; *bclA*	This study
pKJX4	pUC19 containing endoxylanase gene (*xynA*)	[18]
pTJ1-BAN-XynA	6.8 kb, pTJ1-BAN derivative; BAN-fused *xynA* with His6 tag (N-terminus) and FLAG tag (C-terminus)	This study
pTJ1-pelB-BAN-XynA	6.36 kb, pTJ1-BAN derivative; pelB signal peptide, BAN-fused *xynA* with FLAG tag (C-terminus)	This study
pTJ1-pelB-XynA	6.28 kb, pTJ1 derivative; pelB signal peptide, *xynA* with FLAG tag (C-terminus)	This study
pTJ1-BAN-BM3	9.0 kb, pTJ1-BAN derivative; BAN-fused *B. megaterium* monooxygenase (P450-BM3m2) gene with His6 tag (N-terminus) and FLAG tag (C-terminus)	This study
pTJ1-BM3	8.9 kb, pTJ1-BAN derivative; *B. megaterium* monooxygenase (P450-BM3m2) with FLAG tag (C-terminus)	This study

^a^New England Biolabs, Beverly, MA.

^b^Stratagene, Santa Clara, CA.

^c^Pharmacia Biotech, Uppsala, Sweden.

### Plasmids and DNA manipulation

The full gene of the *B. anthracis* RA3 (NCBI accession no. CAD56878.1) *bclA* was synthesized by the *de novo* gene synthesis method (GENEMAKER™; Blue Heron Biotechnology, Bothell, WA). All PCR primers used in this study are listed in Table 2. DNA amplification of the *bclA* gene was performed with primers P1 and P2. The PCR product was digested with *Eco*RI and *Xho*I restriction enzymes, and cloned into pTac99A [24] to yield pTJ1-BAF, which contained the full gene of BclA. Two truncated forms of the *bclA* gene, which contained the N-terminal region (BA-N) or N- and C-terminal fused region (BA-NC) without the repeated region (BA-RD), were synthesized by PCR with the primers P1 and P3, and P4 and P5, respectively. After digestion with *Eco*RI and *Xho*I restriction enzymes, each PCR product was cloned into pTac99A to yield pTJ1-BAN and pTJ1-BANC, respectively. The endoxylanase gene (*xynA*) of *Bacillus* sp. was amplified from pKJX4 [18] as a template by PCR with primers P6 and P7. After digestion with *Nhe*I and *Hin*dIII, the PCR product was cloned into pTJ1-BAN to yield pTJ1-BAN-XynA. The *pelB* signal sequence with the N-terminal region of *bclA* was obtained by PCR with primers P8 and P9, and the PCR product was cloned into the *EcoR*I and *Nhe*I sites of pTJ1-BAN-XynA to yield pTJ1-pelB-BAN-XynA. The *pelB* signal sequence with the endoxylanase gene (*xynA*) without the N-terminal sequence of *bclA* was obtained by PCR with primers P10 and P11 and the PCR product was cloned into the *EcoR*I and *Hind*III sites of pTJ1-BAN to yield pTJ1-pelB-XynA. In order to display P450-BM3 on the cell surface of *E. coli*, the P450 gene was amplified from the monooxygenase variant (BM3m2) of *B. megaterium*[25] by PCR with primers P12 and P13. PCR product was digested with *Nhe*I and *Nco*I and then cloned into pTJ1-BAN to yield pTJ1-BAN-BM3. For cytoplasmic expression of P450, which was used as a negative control, the P450-BM3 gene was amplified by PCR with primers P13 and P14. After digestion with *Eco*RI and *Nco*I, the PCR product was ligated into the pTJ1-BAN expression vector to yield pTJ1-BM3. All PCR was performed with a PCR Thermal Cycler T1 (Biometra, Goettingen, Germany) using a High-Fidelity PCR System (Boeheinger Mannheim, Mannheim, Germany). All DNA manipulations, including restriction digestion, ligation, and agarose gel electrophoresis, were carried out using standard procedures [26].

**Table 2:** Oligonucleotides used for PCR amplification in this study

**No.**	**Primer sequences (5′ → 3′)**^**a**^	**Gene to be amplified**	**Source or references**
P1^b^	G**GAATTC**ATG*CACCACCACCACCACCAC*GCATTTGACCCTAATCTT	Full *bclA* gene	*B. anthracis* RA3
P2	AGTCTAGACTCGAGGCTAGCCCCGGGGGTAGGAAGGGTAAATGG		
P3	CCACCATTTACCCTTCCTACCGGGCCATCCGGACTAGGACTT	*Truncated bclA* gene (NTD)	*B. anthracis* RA3
P4	AAGTCCTAGTCCGGATGGCCCGGTAGGAAGGGTAAATGGTGG	*Truncated bclA* gene (NTD + CTD)	*B. anthracis* RA3
P5	CGTCTA**GACTCGAGGC**TAGC**CCCGG**GAGCAACTTTTTCAATAA		
P6	CAG**GCTAGC**GCTGGCACAGATTACTGGC	*Bacillus* sp. Endoxylanase (XynA)	[18]
P7	CC**AAGCTT**ATTTGTCATCGTCATCTTTATAATCCCACACTGTTACATTAGAACTTC		
P8	A**GAATTC**ATGAAATCCCTATTGCCTACGGCAGCCGCTGGATTGTTATTACTCGCGGCCCA		
P9	ATTGTTATTACTCGCGGCCCAGCCGGCCATGGCGGCTGGCACAGATTACTGGC		
P10	A**GAATTC**ATGAAATCCCTATTGCCTACGGCAGCCGCTGGATTGTTATTACTCGCGGCCCAGCCGGCCATGGCGGCATTTGACCCTAATCTTGTAGG		
P11	T**GCTAG**CCCCGGGGGT		
P12	G**GGCTAGC**ATGACAATTAAAGAAATGCCTCAGCC	*Bacillus megaterium* Monooxygenase (P450 BM3m2)	[19]
P13	GC**CCATG**GCTATTATTTGTCATCGTCATCTTTATAATCCCCAGCCCACACGTC		
P14	GG**GAATTCA**TGACAATTAAAGAAATGCCTCAGCC		

^a^ Restriction enzyme sites are shown in bold.

^b^ The sequence for six histidines is shown in italic.

### Fractionation of outer membrane proteins

Outer membrane proteins were prepared and analyzed as previously described [27,28]. Briefly, after washing cells with 0.5 mL of 10 mM Na_2_HPO_4_ buffer (pH 7.2) twice, cells were disrupted by three cycles of sonication (each for 20 s at 15% of maximum output; High-Intensity Ultrasonic Liquid Processors, Sonics & Material Inc., Newtown, CT). After quick centrifugation (12,000 × *g* for 2 min) to remove partially disrupted cells, membrane proteins and the lipid layer were isolated by centrifugation at 12,000 × *g* for 30 min at 4°C and then, pellets were resuspended in 0.5 mL of 10 mM Na_2_HPO_4_ buffer (pH 7.2) and 0.5% (w/v) sarcosyl solution. After incubation at 37°C for 30 min, an insoluble pellet containing outer membrane proteins was obtained by centrifugation at 12,000 × *g* for 30 min at 4°C. After washing the insoluble pellet with 10 mM Na_2_HPO_4_ buffer (pH 7.2), the outer membrane proteins were resuspended in 50 μL of TE buffer (pH 8.0).

### SDS-PAGE and western blotting analysis

Protein samples were analyzed by electrophoresis on a 10% (w/v) SDS-polyacrylamide gel electrophoresis (SDS-PAGE) gel. For immunodetection of the FLAG-tag fused protein, a monoclonal ANTI-FLAG M2 antibody (Sigma-Aldrich) and goat anti-mouse immunoglobulin G (IgG)-horseradish peroxidase (HRP) conjugate (Invitrogen, Carlsbad, CA) were used. An ECL kit (Amersham ECL Prime Western Blotting Detection Reagent, GE Healthcare) was used for signal detection.

### Measurement of endoxylanase activity

Endoxylanase activity was measured using the 3′,5′-dinitrosalicylic acid (DNS) method [29]. After cultivation, cells were washed twice with 1× phosphate-buffered saline (PBS), and then mixed with 2% (w/v) beech wood xylan solution. The reaction mixture was incubated at 37°C, and the supernatant of the mixture was sampled at time intervals by centrifugation for 10 min at 12,000 ×*g* and room temperature. The supernatant samples were mixed with 3 times the volume of a DNS solution containing 7.5 g 3,5-dinitrosalicylic acid (DNS), 14 g NaOH, 216.1 g Rochelle salt, 5.4 mL phenol and 5.9 g Na_2_S_2_O_5_ per liter. After boiling for 10 min, samples were cooled to room temperature for 5 min, and then the absorbance of the reactant was detected by a spectrophotometer at 550 nm. One unit (U) of endoxylanase activity was defined as the amount of enzymes capable of producing 1 μmol of reducing sugar per min. The specific activity was defined as the endoxylanase activity for the amount of cells with an OD_600_ of 2.0.

### Measurement of monooxygenase (P450 BM3m2) activity

After cultivation, cells were harvested by centrifugation at 6,000 rpm for 10 min. Cell pellets were washed twice with 100 mM potassium phosphate buffer (pH 7.4) and the cells were concentrated in the same buffer to a calculated final OD_600_ of 100. The activity of the displayed P450 was determined by analyzing the dealkylation of 7-epoxycoumarin to 7-hydroxycomuarin as described previously [25]. The prepared cells were mixed with 7-ethoxycoumarin (1 mM) and NADPH (0.5 mM) in 100 mM potassium phosphate buffer (pH 7.4). The reaction was performed at room temperature for 30 min, the product (7-hydroxycomuarin) was quantified using a multi-well plate fluorometer (VICTOR ×3, Perkin-Elmer, Waltham, MA) with excitation wavelength at 405 nm and emission wavelength at 460 nm.

### Fluorescence microscopy

After cultivation, cells (1 mL) were washed with 1× PBS and resuspended in 1× PBS supplemented with 3% (w/v) bovine serum albumin (BSA; Sigma-Aldrich). The cells were first incubated with the rabbit anti-FLAG probe antibody conjugated with FITC (Invitrogen) at a dilution of 1:250 for 1 h. Cells were washed 2 times with a PBS solution to remove the unbound probes. Finally, cells were mounted on a poly-l-lysine coated microscopic slide and examined by confocal microscopy (Carl Zeiss LSM510 META, Jena, Germany). Samples were excited by a 488 nm argon laser, and images were filtered by a long pass 505 nm filter. Images were acquired with Carl Zeiss LSM 510 software (version 4.2.rk).

## Competing interests

The authors declare that they have no competing interests.

## Authors’ contributions

TJP designed and performed most experiments, and analyzed data and drafted the manuscript. NSH participated in the display of BclA cloning, and SSY and JHP participated in the display of endoxylanase and P450. KJJ and SYL coordinated the study and contributed to the experimental design, data interpretation, and reviewing the manuscript. All authors have read and approved the final manuscript.

## Supplementary Material

Additional file 1: Figure S1 Multiple alignments of several BclA proteins and schematic representation of the BclA protein consisting of GXX triplet motifs. Click here for file

Additional file 2: Figure S2 SDS-PAGE analysis for the expression of lipase (Lip1) in three different BclA anchoring systems. Click here for file

Additional file 3: Figure S3 Display of endoxylanase on the cell surface. Click here for file

Additional file 4: Figure S4 Endoxylanase activity. Click here for file
