# Supplementary material for: Surface display of recombinant proteins on Escherichia coli by BclA exosporium of Bacillus anthracis
Source: Microb Cell Fact. 2013 Sep 22;12:81. doi: 10.1186/1475-2859-12-81 (PMC3850424; doi:10.1186/1475-2859-12-81)
Supplement: Additional file 1: Figure S1 — Multiple alignments of several BclA proteins and schematic representation of the BclA protein consisting of GXX triplet motifs. [file 1475-2859-12-81-S1.docx]

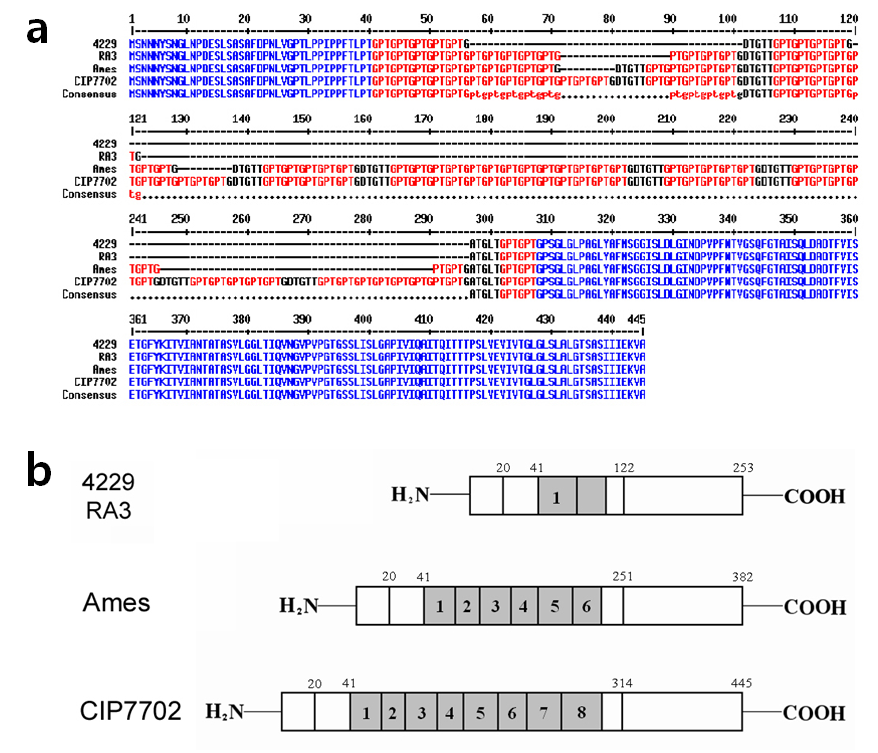


**Figure S1. Multiple alignments of several BclA proteins and schematic representation of the BclA protein consisting of GXX triplet motifs.** (a) Multiple alignment of BclA amino acid sequences. Blue amino acids, N- and C-terminal conserved region; Red amino acids, GPT triplet motif; Black amino acids, GXX triplet, (b) GXX triplet motifs are in shaded in grey. 4229, *Bacillus anthracis* ATCC4229 strain (NCBI accession number CAD56880); RA3, *B. anthracis* RA3 strain (NCBI accession number CAD56878); Ames, *B. anthracis* Ames strain (NCBI accession number CAD56869); CIP7702, *B. anthracis* Sterne CIP7702 strain (NCBI accession number CAD56870).
