# Supplementary material for: Surface display of recombinant proteins on Escherichia coli by BclA exosporium of Bacillus anthracis
Source: Microb Cell Fact. 2013 Sep 22;12:81. doi: 10.1186/1475-2859-12-81 (PMC3850424; doi:10.1186/1475-2859-12-81)
Supplement: Additional file 2: Figure S2 — SDS-PAGE analysis for the expression of lipase (Lip1) in three different BclA anchoring systems. [file 1475-2859-12-81-S2.docx]

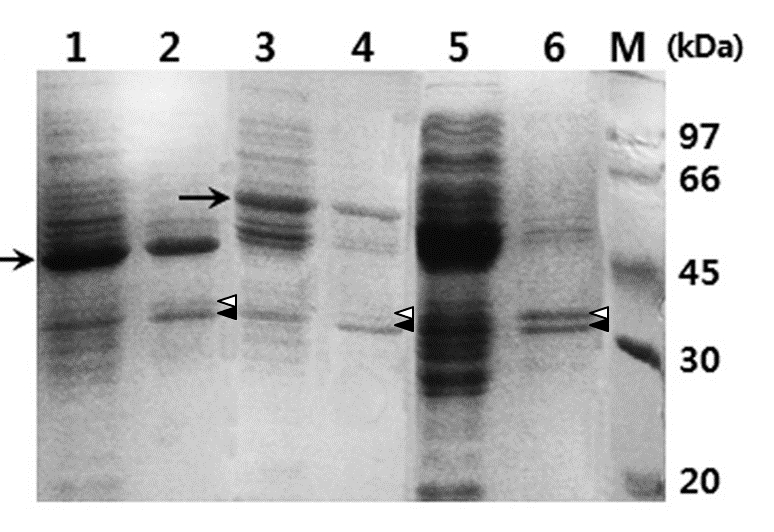


**Figure S2.** **SDS-PAGE analysis for the expression of lipase (Lip1) in three different BclA anchoring systems***.* Lane M, molecular protein size marker (kDa); Lanes 1 and 2, *E. coli* JM109 harboring pTac99a; Lanes 3 and 4, *E. coli* JM109 harboring pTJ1-BAN-Lip1; Lanes 5 and 6, *E. coli* JM109 harboring pTJ1-BANC-Lip1; Lanes 7 and 8, *E. coli* JM109 harboring pTJ1-BAF-Lip1. Lanes 1, 3, and 5, total proteins fraction; Lanes 2, 4 and 6, outer membrane proteins fraction. In lanes 1 and 3, arrows indicates BAN-Lip1 (46.4 kDa) and BANC-Lip1 (61.9 kDa), respectively. Closed and open arrowheads indicate the OmpA and OmpC proteins bands in outer membrane proteins fraction, respectively. We want to emphasize that the results obtained with lipase were rather significantly variable during multiple experiments due to unknown reasons.
