# Supplementary material for: Surface display of recombinant proteins on Escherichia coli by BclA exosporium of Bacillus anthracis
Source: Microb Cell Fact. 2013 Sep 22;12:81. doi: 10.1186/1475-2859-12-81 (PMC3850424; doi:10.1186/1475-2859-12-81)
Supplement: Additional file 3: Figure S3 — Display of endoxylanase on the cell surface. [file 1475-2859-12-81-S3.docx]

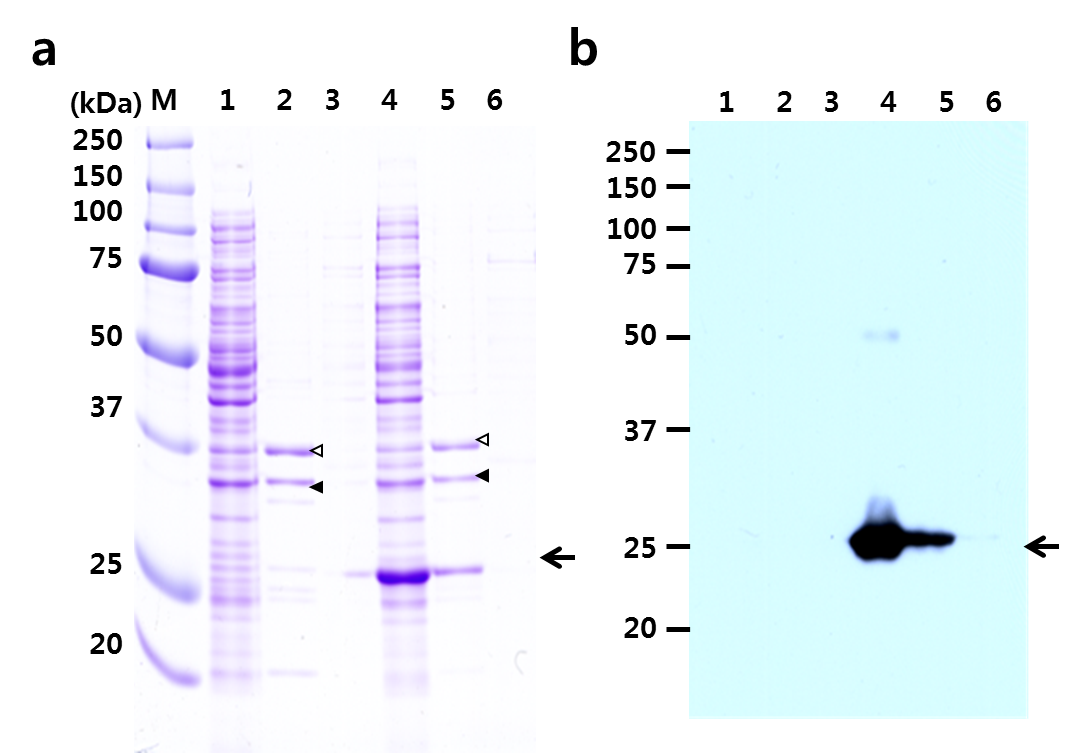


**Figure S3. Display of endoxylanase on the cell surface.** (a) SDS-PAGE analysis and (b) western blotting analysis of *E. coli* displaying BAN-fused XynA. Lane M, molecular weight size markers; Lanes 1 to 3, *E. coli* harboring pTJ1-BAN (without XynA); lane 4 to 6, *E. coli* harboring pTJ1-BAN-XynA. Lanes 1 and 4, total proteins; Lanes 2 and 5, outer membrane protein fractions; Lanes 3 and 6, culture supernatants. The culture supernant analysis, 10 times concentrated samples were loaded on PAGE gel. Arrows indicate the BAN-fused endoxylanase. Closed and open arrowheads indicate the OmpA and OmpC proteins bands in outer membrane proteins fraction, respectively. In (b), one faint band is shown at ~50 kDa size. We think this is not a dimerized form of BAN-fused endoxylanase (~24 kDa) but just an artifact by non-specific binding because SDS-PAGE experiment was done in reducing condition with boiling, and so the dimerized form cannot be detected.
