# Supplementary material for: Surface display of recombinant proteins on Escherichia coli by BclA exosporium of Bacillus anthracis
Source: Microb Cell Fact. 2013 Sep 22;12:81. doi: 10.1186/1475-2859-12-81 (PMC3850424; doi:10.1186/1475-2859-12-81)
Supplement: Additional file 4: Figure S4 — Endoxylanase activity. [file 1475-2859-12-81-S4.docx]

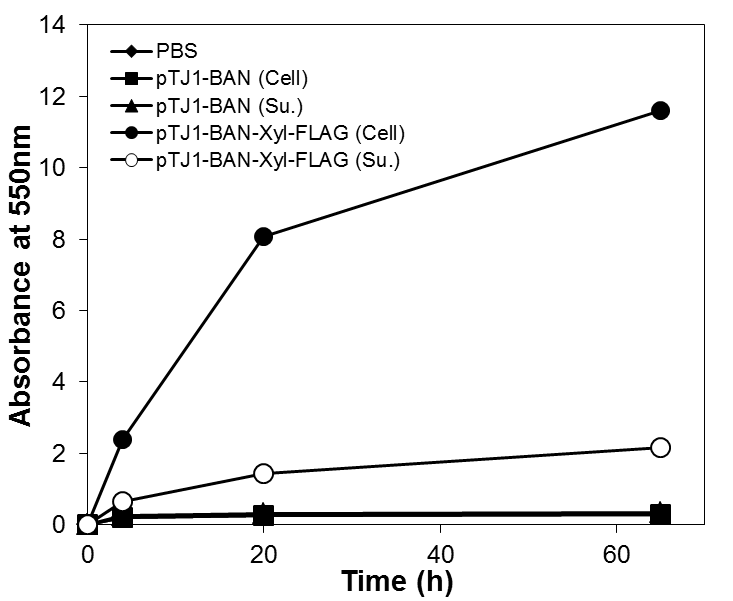


**Figure S4. Endoxylanase activity.** Symbols:⧫, PBS solution as a negative control; ■, cells harboring pTJ1-BAN; ▲, Culture supernatant of cells harboring pTJ1-BAN; ●, cells harboring pTJ1-BAN-XynA ; ○, Culture supernatant of cells harboring pTJ1-BAN-XynA.
